# Selective anticancer activity of a hexapeptide with sequence homology to a non-kinase domain of Cyclin Dependent Kinase 4

**DOI:** 10.1186/1476-4598-10-72

**Published:** 2011-06-13

**Authors:** Hilmar M Warenius, Jeremy D Kilburn, Jon W Essex, Richard I Maurer, Jeremy P Blaydes, Usha Agarwala, Laurence A Seabra

**Affiliations:** 1 Visiting Professor of Anti-Cancer Drug Development School of Chemistry, University of Southampton, Highfield, Southampton, SO17 1BJ, UK; 2 School of Chemistry, University of Southampton, Highfield, Southampton, SO17 1BJ, UK; 3 Faculty of Medicine, University of Southampton, Southampton, SO16 6YD, UK; 4 Post Doctoral Fellow, Medical and Molecular Genetics. The Institute of Biomedical Research University of Birmingham, Edgbaston, Birmingham, UK; 5 School of Biological and Chemical Sciences, Queen Mary University of London, Mile End Road, London E1 4NS, UK

**Keywords:** Cdk4, non-kinase, proteomic, PRGPRP, programmed cell death, selective anticancer, broad spectrum

## Abstract

**Background:**

Cyclin-dependent kinases 2, 4 and 6 (Cdk2, Cdk4, Cdk6) are closely structurally homologous proteins which are classically understood to control the transition from the G1 to the S-phases of the cell cycle by combining with their appropriate cyclin D or cyclin E partners to form kinase-active holoenzymes. Deregulation of Cdk4 is widespread in human cancer, *CDK4 *gene knockout is highly protective against chemical and oncogene-mediated epithelial carcinogenesis, despite the continued presence of *CDK2 *and *CDK6*; and *o*verexpresssion of Cdk4 promotes skin carcinogenesis. Surprisingly, however, Cdk4 kinase inhibitors have not yet fulfilled their expectation as 'blockbuster' anticancer agents. Resistance to inhibition of Cdk4 kinase in some cases could potentially be due to a non-kinase activity, as recently reported with epidermal growth factor receptor.

**Results:**

A search for a potential functional site of non-kinase activity present in Cdk4 but not Cdk2 or Cdk6 revealed a previously-unidentified loop on the outside of the C'-terminal non-kinase domain of Cdk4, containing a central amino-acid sequence, Pro-Arg-Gly-Pro-Arg-Pro (PRGPRP). An isolated hexapeptide with this sequence and its cyclic amphiphilic congeners are selectively lethal at high doses to a wide range of human cancer cell lines whilst sparing normal diploid keratinocytes and fibroblasts. Treated cancer cells do not exhibit the wide variability of dose response typically seen with other anticancer agents. Cancer cell killing by PRGPRP, in a cyclic amphiphilic cassette, requires cells to be in cycle but does not perturb cell cycle distribution and is accompanied by altered relative Cdk4/Cdk1 expression and selective decrease in ATP levels. Morphological features of apoptosis are absent and cancer cell death does not appear to involve autophagy.

**Conclusion:**

These findings suggest a potential new paradigm for the development of broad-spectrum cancer specific therapeutics with a companion diagnostic biomarker and a putative functional site for kinase-unrelated activities of Cdk4.

## Background

Cdk4 has been actively pursued, over the last two decades, as a promising anticancer drug target [1,2] based on its role in cell cycle control [3] and its widespread deregulation in a multiplicity of different tumours [4]. Single agent activity of cyclin dependent kinase inhibitors in general has, however, been disappointing, with low percentages of objective responses [5], and no Cdk inhibitor has yet been approved as an anticancer drug [6] In particular, the specific Cdk4 inhibitor, flavopiridol, yielded no objective responses in phase II studies of metastatic melanoma, endometrial adenocarcinoma and multiple myeloma [7] when used as a single agent. Following encouraging animal studies [8], newer Cdk4 kinase inhibitors such as PD 0332991, are now entering clinical trials in combination with agents of already proven activity such as bortezomib rather than as single agents. Nonetheless, *CDK4 *gene knockout in mice completely abrogates chemically induced epidermal carcinogenesis [9], without effect on normal skin keratinocyte proliferation, despite the continuing presence of Cdk2 and Cdk6. Conversely low levels or absence of Cdk6 do not prevent the in-vitro growth of human breast cancer cell lines [10]. Additionally, ablation of *CDK4 *[11] but not of *CDK2 *[12] inhibits myc-mediated oral tumorigenesis. Furthermore, overexpression of Cdk4 but not cyclin D1 promotes mouse skin carcinogenesis [13], whilst elevated Cdk2 activity, despite inducing keratinocyte proliferation, is not tumorogenic [14]. Cdk4 would therefore appear to be the key cyclin-dependent kinase for both chemical and oncogene promoted epithelial carcinogenesis. The poor activity of drugs directed against Cdk4 kinase is thus surprising.

The paradigm relating cell cycle control, cyclin-dependent kinases and cancer has changed from our classical understanding, however, with reappraisal of the mandatory requirement of Cdk2, Cdk4 or Cdk6 for normal cell division [15]. Moreover, Cdk4 and Cdk6 have been shown to differ functionally from one another in several respects [16-19] and Cdk4 has been reported to be closely co-expressed with Cdk1 in a wide range of human cancers *in-vitro *and in malignant melanoma in the clinic but not in normal diploid fibroblasts or keratinocytes [20]. Evidence is also now accumulating that Cdk4 can exhibit kinase-unrelated activities. Direct interaction of Cdk4 with Myo-D to restrict myoblast differentiation in the absence of Cdk4 kinase activity has been reported [21] and mutant, kinase-dead, *CDK4-N158D *upregulation in TIG3 cells can induce p16INK4 expression equally as well as kinase-active, wild-type CDK4 [22]. Recently, resistance to EGFR tyrosine kinase inhibitors, in PCMM2 prostate cancer cells, has been reported to be related to a kinase-independent function of EGFR which prevents autophagy by maintaining intracellular glucose levels [23]. Possibly the apparent discordance between results from *CDK4 *knockout cancer models, and Cdk4 kinase inhibitor studies could stem from such critical kinase-independent activities of Cdk4.

Divergent roles for Cdk4 and Cdk6 and evidence of kinase-unrelated activities in cyclin-dependent and other kinases along with the differences between Cdk4 and Cdk2 or Cdk6 in promoting carcinogenesis, prompted a search for a functional site of kinase-independent activity specific to Cdk4 but not Cdk2 or Cdk6. Binding sites for proteins intrinsic to classical Cdk4 kinase activity all lie predominantly within the N'-terminal 2/3 of the Cdk4 protein. Structural studies of the whole Cdk4, Cdk6 and Cdk2 molecules were therefore carried out to search for a potential kinase-independent functional site within the C'-terminal domain of Cdk4, not shared by Cdk6 or Cdk2. These studies revealed a previously-undescribed, proline/arginine rich, 12 amino-acid site, FPPRGPRPVQSV, on the outside surface of Cdk4.

Proteomic expression levels of Cdk1 and Cdk4 are closely correlated in human cancers but not normal cells [20] and have been shown to spontaneously go up and down together from experiment to experiment in several human cancer cell lines [24]. Cdk4 overexpression following transfection is accompanied by concomitant increase in Cdk1 expression in RAMA37 cells [20] and disruption of Cdk1/Cdk4 co-expression can be observed in human cancer cells undergoing spontaneous cell death [25]. Possibly the FPPRGPRPVQSV region of Cdk4 might be involved in control of Cdk1 expression. Such a mechanism could be vulnerable to competitive inhibition, disrupting the Cdk1/Cdk4 co-relationship and potentially destabilising malignant cells. To test this possibility, human cancer cells were exposed to peptide fragments of varying length derived from the FPPRGPRPVQSV sequence and the effects on cell proliferation and relative Cdk1/Cdk4 levels monitored.

## Results

### Proteomic co-expression of Cdk1/Cdk4 in human cancer cell lines is not seen with Cdk1/Cdk2 or Cdk1/Cdk6

**Figure 1:**
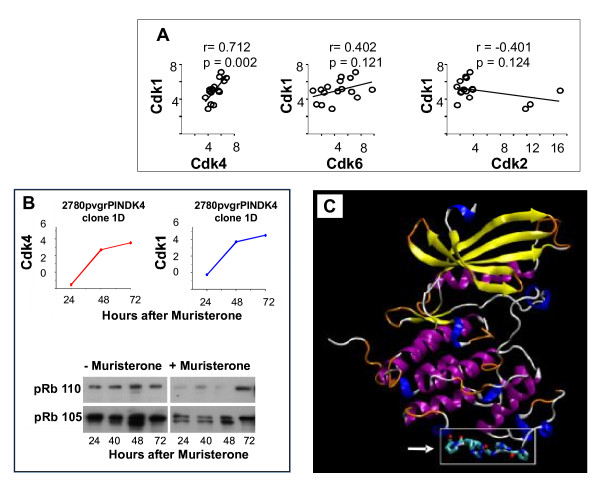
**A potential protein-protein non-kinase functional binding site in Cdk4 not present in Cdk2 or Cdk6**. **A **- Relative proteomic expression by Western blotting, of Cdk4, Cdk6 and Cdk2 compared to Cdk1 on 16 human in vitro cancer cell lines of widely differing histological subtype by quantitative Western blotting as previously described [20]. Primary antibodies were Cdk1 - mouse monoclonal sc-54 (1/250), Cdk2 - (M2) rabbit polyclonal sc-163 (1/250), Cdk4 - rabbit polyclonal sc-260 (1/250) Cdk6 - rabbit polyclonal sc-260 at 1/250, (see methods). **B **- Upper panels: Endogenous Cdk1 follows a similar time course and increase in proteomic expression as exogenous Cdk4 induced by 1 μM muristerone in 2780 pvrg-pINDK4 clone 1D cells (see methods). Lower panels Western blotting for total retinoblastoma protein (pRb 105) and hyperphosphorylated retinoblastoma protein (p110) following exposure of 2780 pvrgpINDK4 clone 1D cells to 1 μM muristerone. **C **- Location of the PRGPRP central hexameric amino acid sequence (residues 249 - 260) of FPPRGPRPVQS within an externalised loop in the C' terminal region, distant from the classically described kinase domain of Cdk4.

Figure 1 compares proteomic expression of Cdk1 to Cdks 2, 4 and 6 using linear regression analysis across 16 human in-vitro cell lines, covering a wide range of histological subtypes. Only Cdk4 has a highly significant co-expressive relationship with Cdk1.

Previous experiments have demonstrated a causal relationship between Cdk4 and Cdk1 expression in 10 positive clones from RAMA37 cells transfected with *CDK4 *cDNA [20]. To confirm this relationship in a different system, an ecdysone-inducible *CDK4 *expression vector was introduced to 2780 human ovarian carcinoma cells and positive transfectants selected. Induction of exogenous Cdk4 expression by 1 μM muristerone (Figure 1B) in this system, was accompanied by contemporaneous increase in Cdk1 protein. In addition there was no change in the phosphorylation status of the retinoblastoma protein.

### Modelling studies

Prior to recent reports [4,26] of a crystal structure for Cdk4, it was necessary to use the considerable sequence homology across the Cdk protein family to produce a comparative model. Related sequences and important regions within the Cdk4 molecule were identified, a model of Cdk4 based on experimentally determined structures of Cdk6 and Cdk2 was built and regions in the Cdk4 model that might provide support for a kinase-unrelated binding site were sought. Although the protein data bank contained a model structure for Cdk4 (1LD2), an independent model was built which might provide advantageous additional insights. Sequence alignment of Homo Sapiens Cdk4 with a range of Cdk4 sequences from mammalian and non-mammalian organisms was initially performed.

For the N'-terminal half of the non-mammalian sequences of Cdk4, corresponding to the first domain and responsible for the majority of its function, the sequence is well conserved. However, the C'-terminal third of the sequence showed considerable variability. In particular, our attention was drawn to the FPPRGPRPVQSV sequence in Homo Sapiens Cdk4 which showed little or no conservation beyond mammalian species.

Sequence alignment of Cdk4, 6, and 2, showed considerable conservation between all three proteins, as expected. The 12mer segment previously identified in the alignment of mammalian and non-mammalian Cdk4 sequences, however, exhibited very little homology with either Cdk6 or Cdk2 (also apparent in data from an independent Cdk modelling exercise [27]), again suggesting that it might have a unique functional role. In addition to Cdk4, a search of the protein database for similar sequences to "FPPRGPRPVQSV" only returned Ras GTPase-activating protein-binding protein 2 for which there is, at present, no structural data.

An homology model of Cdk4 was built using a combination of Cdk2 and Cdk6 protein structures as templates. Missing segments of the sequence, and in some cases, crystal packing contacts, in the majority of structures used, may have affected the conformations adopted. Thus the quality of any derived homology model could be suboptimal. For the purposes of this study, however, it was reasonable to expect the model to provide reliable information on the tertiary structure, the location of amino acids within the structure, and whether or not they are solvent accessible. Five homology model structures of Cdk4 were initially constructed, with that using the Cdk6 template 1BLX being considered the most reliable (Table 1). The loop containing the previously identified 12mer (the central hexamer of which, PRGPRP, is shown in all-atom detail in cartoon representation [28] (Figure 1C) is solvent accessible in all models and varies only slightly in conformation. This sequence is situated in domain 2 (amino acid residues 249 - 260) at the furthest point away from domain 1 and differs from that of Cdk2 and Cdk6 in being rich in arginine and proline. The bioinformatics and structural evidence together, suggests this sequence as a plausible candidate for a kinase-independent functional site. Our structural modelling is validated by the recently published crystal structure data [4,26].

**Table 1:** Quality and accuracy scores for the built models

MODEL	Model 1	Model 2	Model 3	Model 4	Model 5
Template	1BLX (CDK6)	1G3N (CDK6)	Base template 1BLX, Variable regions differing by more than 2.0 Å rmsd modelled from 1G3N, 1BI8, 1BI7 and 1JOW (all CDK6)	Base template 1BLX, Variable regions differing by more than 2.0 Å rmsd. modelled from 1HCL (CDK2)	Base template 1BLX, Variable regions differing by more than 2.0 Å rmsd. modelled from 1GII (CDK2)

Threading score	165.6	158.2	151.0	128.8	99.7

Molecular mechanics energy (kJ mol^-1^)	-12203.3	-12526.1	-12182.5	-11900.3	-11795.5

RMS deviation from 1BLX (in Å)	0.48	0.88	0.62	0.67	0.65

*Structure Z-scores, positive is better than average*					

2^nd ^generation packing quality	-1.093	-0.868	-0.964	-1.090	-1.228

Ramachandran plot appearance	-2.573	-3.374	-2.837	-2.965	-3.104

*χ*-1/*χ*-2 rotamer quality	-1.148	-1.470	-1.340	-0.955	-0.968

Backbone conformation	-6.485	-5.201	-5.637	-7.016	-7.564

*RMS Z-scores,should be close to 1*					

Bond lengths	0.655	0.645	0.652	0.657	0.668

Bond angles	1.187	1.176	1.183	1.168	1.181

Omega angle restraints	1.354	1.159	1.413	1.478	1.396

Side chain planarity	1.608	1.667	1.494	1.277	1.292

Improper dihedral distribution	0.883	0.882	0.907	0.879	0.865

Inside/Outside distribution	1.019	1.038	1.025	1.043	1.051

A high threading score indicates a better fit of the sequence in the structure. A low molecular mechanics energy indicates a more relaxed structure.

Structural Z-scores less than -2.0 indicate problems in the model, scores less then -4.0 indicates serious errors. RMS Z-scores should be close to 1.0.

### An isolated hexapeptide, homologous with the central sequence of the FPPRGPRPVQ external Cdk4 loop, kills human RT112 bladder cancer cells but not normal diploid human fibroblasts

**Figure 2:**
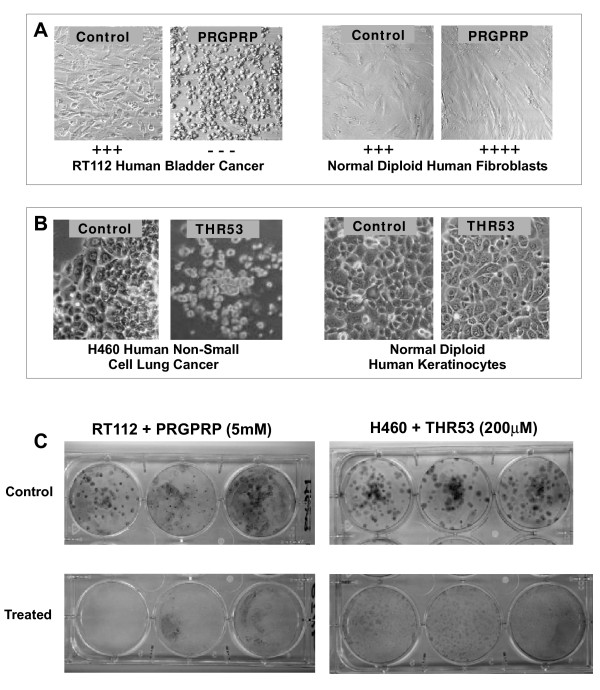
**Selective in-vitro cell killing of human cancer cells by exposure to linear N'-capped PRGPRP-amide and the derived cyclic amphiphilic analogue, THR53**. (cyc-[FPPRGPRPVKLALKLALK]) **A **Photomicrographs illustrating typical in-vitro morphological appearances of RT112 human bladder cancer cells (Left hand panels) and normal human fibroblasts (Right hand panels) following exposure to 5.0 mM PRGPRP (Ac-Pro-Arg-Gly-Pro-Arg-Pro-NH_2_) or control nonsense peptide. PRGPRP causes complete necrosis of RT112 bladder cancer cells but increases the confluent cell density of normal diploid fibroblasts (Magnification ×40). **B**. Photomicrographs illustrating typical in-vitro morphological appearances of NCI-H460 human non-small cell lung cancer cells (Left hand panels) and normal diploid human keratinocytes (Right hand panels) following exposure to 200 μM THR53 or control nonsense peptide. (Magnification × 40) **C**. Macroscopic appearances of clonogenic assay tissue culture wells of RT112 human bladder cancer cells exposed to 5 mM PRGPRP (left hand panels) and H460 human non-small cell lung cancer cells exposed to 200 μM THR53 (right hand panels).

A broad search for possible biological activity associated with the FPPRGPRPVQSV peptide sequence was initially instituted by screening human bladder cancer cell lines for potential in-vitro morphological changes, compared to control cultures of normal diploid human fibroblasts at 4-8 passages from primary culture as controls. A range of linear N'-capped, synthetic peptides matching 4 - 10 amino acid lengths of the sequence FPPRGPRPVQSV were prepared as amides (Polypeptide Ltd). The peptides were dissolved, over a range of concentrations, in Ham's F12 + 10% FCS and introduced in 100 μL quantities to microwells containing 500 RT112 bladder cancer cells, or normal diploid human fibroblasts, in 200 μL of Ham's F12 + 10% FCS plated 24 hours earlier on 96 well plates. The whole FPPRGPRPVQ peptide (Table 2,I) was found to be selectively toxic to RT112 human bladder cancer cells, at a concentration of 1.0 mM, with partial sparing of normal fibroblasts. A hexapeptide PRGPRP and a pentapeptide RGPRP homologous to the central region of FPPRGPRPVQ, showed greater selective toxicity to RT112 human bladder cancer cells whilst sparing normal diploid fibroblasts at concentrations of 5 mM (Table 2,II). Morphological analysis demonstrated PRGPRP stimulates normal fibroblast growth whilst showing high toxicity against RT112 cells (Figure 2A). Figure 2C shows representative colony survival assays of PRGPRP-treated RT112 cells. Shorter linear peptides such as RGPR were inactive and cells exposed to them grew with similar morphological appearances to untreated controls. (Table 2,V). Similar results (not shown) were obtained with the MGHU1 human bladder cancer cell line.

**Table 2:** In-vitro Survival of RT112 Human Bladder Cancer and Normal Diploid Human Fibroblasts

			Cell Viability	
	**Compound**	**Dose**	**Human Bladder Cancer**	**Normal Diploid Fibroblasts**

I.	FPPRGPRPVQ	1.0 mM	+/-	+ +/-

II. (THR1)	PRGPRP	5.0 mM	- - -	+ + + + +

III.	PRGPR	5.0 mM	- - -	+ + +

IV.	RGPRP	5.0 mM	+/-	+ + +

V.	RGPR	5.0 mM	+ + +	+ + +

VI.	PRRPGP	5.0 mM	+ + +	+ + +

VII.	PEGPRP	5.0 mM	+ + +	+ + +

VIII.	PRGPEP	5.0 mM	+ + +	+ + +

IX.	PEGPEP	5.0 mM	+ + +	+ + +

(+++ = same morphological appearance as untreated contros, --- = total morphological cell death. See Figure 2A for typical examples)

### PRGPRP cell killing is specific for cancer cells and is amino-acid sequence dependent

**Figure 3:**
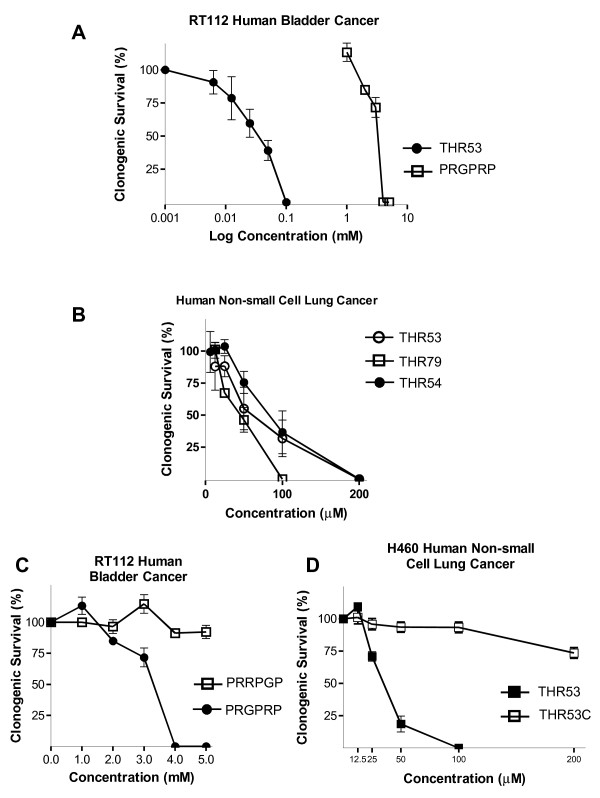
**Clonogenic cell survival assays of RT112 human bladder cancer cells and H460 human non-small cell lung cancer cells exposed to PRGPRP vs PRRPGP as linear hexapeptides or in cyclic amphiphilic cassettes**. **A**. Comparison of response of RT112 human bladder cancer cells to PRGPRP and THR53. **B**. Clonogenic cell survival of H460 human non-small cell lung cancer cells to THR53 (cyc-[FPPRGPRPVKLALKALAK]); THR54 (cyc-[PRGPRPVALKLALKLAL]) and THR79 (cyc-[PRGPRPvalklalkalal]). (Capitals signify L-amino acids, lower case signify D-amino-acids). **C**. Comparative clonogenic cell survival of RT112 human bladder cancer cells exposed to the linear end-capped hexapeptides PRGPRP or PRRPGP. **D**. Comparative clonogenic cell survival of H460 human non-small cell lung cancer cells exposed to the cyclic amphiphilic peptides: THR 53 (cyc-[FPPRGPRPVKLALKALAK]) or THR 53C (cyc-[FPPRRPGPVKLALKALAK]).

The amino acid sequence within the hexapeptide is critical for cancer cell lethality. Thus transposing one of the two arginines, to give PRRPGP rather than PRGPRP, markedly diminishes RT112 human bladder cancer cell killing (Table 2,VI). Moreover, PEGPRP and PRGPEP, in which the arginines were substituted by glutamic acids, both lacked cytotoxic activity against RT112 and had no effect on normal diploid human fibroblasts (Table 2,VII-IX). The selective anticancer activity of PRGPRP as compared to PRRPGP was confirmed by clonogenic assays on RT112 (Figure 3C).

### Cyclic amphiphilic constructs containing the PRGPRP amino-acid sequence show specific activity at micromolar concentrations and a shorter time to cell death

**Figure 4:**
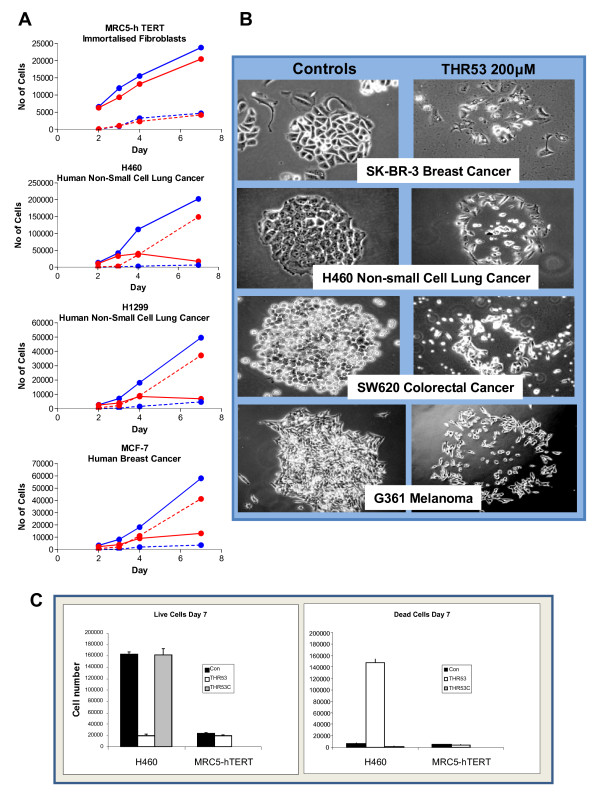
**Time course of cell death and morphological appearances of a range of cancers of different histologies exposed to 200 μM THR53**. **A**. Time course of loss of viability in H460 human non-small cell lung cancer, H1299 human non-small cell lung cancer and MCF-7 human breast cancer cells exposed to 200 μM THR53. MRC5-hTERT cells were unaffected by 200 μM THR53. Viability assessed by Trypan Blue exclusion of MRC5-hTERT, H460, H1299 and MCF-7 cells in the presence or absence of the compound, trypsinised off and stained at the appropriate time point. Each data point is the mean of results from triplicate wells (Solid lines - Live cells, Broken lines - Dead Cells; Blue - Control, Red - THR53 200 μM). **B**. Morphgological appearances of ubiquitous response to 200 μM THR53 of: SK-BR-3 human breast cancer, H460 human non-small cell lung cancer, SW620 human colorectal cancer and G361 malignant melanoma cells. **C**. Side by side quantification of H460 and MRC-5 cell viability assays at a single, 7 day, time point comparing THR53 (PRGPRP in cyclic amphiphilic cassette) and THR53C (PRRPGP in cyclic amphiphilic cassette) after initial exposure to compounds at a concentration of 200 μM.

**Figure 5:**
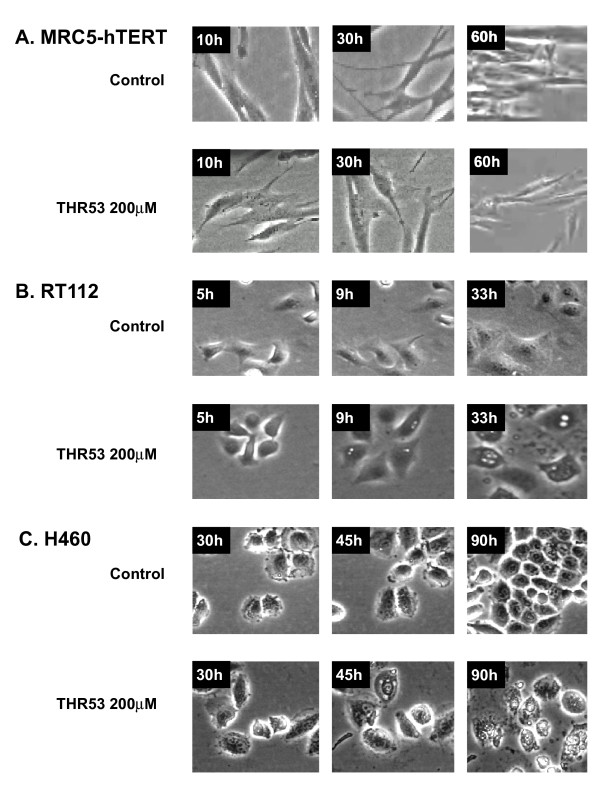
**Time Lapse Photomicrography of MRC5-hTERT, RT112 human bladder cancer and H5460 human non-small cell lung cancer following exposure toTHR53**. Cells were incubated in Ham's F12 + 10% FCS (control medium) or medium + 200 μM THR53. Morphological changes of vacuolation + cellar swelling are seen in RT112 at 33 hours after exposure to THR53 and in H460 at 90 hours after exposure to THR53. There was no evidence of cell blebbing or fragmentation. (see also videos)

Attempts to limit proteolysis and improve cell uptake, by incorporating the hexameric peptide sequence into cyclic peptide cassettes were undertaken, initially without success (Table 3,X-XIII). Linear constructs attaching PRGPRP to a known cell-internalising amphiphilic sequence [29] were also unsuccessful (Table 3,XIV and 3XV). Combining the two approaches to create cyclic amphiphilic peptide cassettes, however, resulted in a number of peptides with a fifty- to a hundred-fold improvement in selective cancer cell cytotoxicity (Table 4,XVI, XVIII, XIX & Figures 2C &3A). Cell killing by THR53, THR54 and THR79, of the RT112 human bladder cancer and H460 human non-small lung cancer cell lines, was seen at concentrations of 50 μM - 200 μM (Table 4 & Figures 3A, B) and, for the majority of cell lines, reached a maximum within 4 days (Figures 4A, 5C). Selective toxicity to these cancer cell lines, and not normal fibroblasts, was retained (Table 4).

**Table 3:** In-vitro Survival of H460 Human Non-small Cell Lung Cancer and Normal Diploid Human Fibroblasts Following Exposure to Cyclic Non-Amphiphilic or Linear Amphiphilic Analogues of PRGPRP.

			Cell Viability	
	**Compound**	**Dose**	**Human Non-small Cell Lung Cancer**	**Normal Diploid Fibroblasts**

X.	Cyc-[AAAGGGPRGPRPGGGAAA]	200 μM	+ + +	+ + +

XI.	Cyc-[GGGGGGPRGPRPGGGGGG]	200 μM	+ + +	+ + +

XII.	Cyc-GGGGGGPRGPRPGGGGGG]	200 μM	+ + +	+ + +

XIII.	Cyc-[AAGPGGPRGPRPGGPGAA]	200 μM	+ + +	+ + +

XIV.	Linear FPPRGPRPVKLALKLALK	200 μM	+ + +	+ + +

XV	Linear PRGPRPVALKLALKLAL	200 μM	+ + +	+ + +

**Table 4:** In-vitro Survival of H460 Human Non-small Cell Lung Cancer and Normal Diploid Human Fibroblasts Following Exposure to Cyclic Amphiphilic Analogues of PRGPRP.

			Cell Viability	
	**Compound**	**Dose**	**Human Non-small Cell Lung Cancer**	**Normal Diploid Fibroblasts**

XVI. (THR53)	Cyc-[FPPRGPRPVKLALKLALK]	200 μM	_ _ _	+ + +

XVII. (THR53C)	Cyc-[FPPRRPGPVKLALKLALK]	200 μM	+ + +	+ + +

XVIII. (THR54)	Cyc-[PRGPRPVALKLALKLAL]	100 μM	_ _ _	+ + +

XIX. (THR79)	Cyc-[PRGPRPvalklalklal]	50 μM	_ _ _	+ + +

XX.	Cyc-[PR(Me)GPRPVALKLALKLAL]	200 μM	+ +/-	N/D

XXI.	Cyc-[PRGPR(Me)PVALKLALKLAL]	200 μM	++ +	N/D

XII.	Cyc-[PR(Me)GPR(Me)PVALKLALKLAL]	200 μM	+ + +	N/D

### Importance of arginines in the cancerocidal activity of PRGPRP-containing cyclin amphiphilic peptides

The previously observed, arginine-related, structure/function relationship to PRGPRP at 5 mM (Table 2,II vs. 2,VII & Figure 3C) was retained in the cyclic amphiphilic compounds (Compare survival of H460 cells exposed to THR53 or THR53C Figures 3D, 4C). Additionally N-mono-methylation within the guanidium moiety of the arginines (Table 4,XX-XXII) removed the cytotoxic activity of THR54 against H460 cells.

### THR53 kills a wide range of human cancer cells but not MRC5-hTERT immortalised fibroblasts or human diploid keratinocytes

After exposure to 200 μM THR53, widely histologically different human cancer cell lines all exhibited closely similar morphological changes at 5 days (Figure 4B), followed by complete lethality in clonogenic assays at 15 days. The appearances of the disintegrating colonies in Figure 4B were not dissimilar to those of "starbursts" earlier reported [25] in human in vitro cancer cell colonies undergoing spontaneous cell death. Table 5 lists 11 cell lines with complete response to 200 μM THR53. This relatively homogeneous therapeutic response to the same critical dose of THR53 markedly contrasts with the wide range of variability of in-vitro dose response of different cancer cell lines typically seen with conventional chemotherapeutic agents [30] In addition to showing lack of toxicity towards normal human diploid fibroblasts, THR53 and related cyclic amphiphilic PRGPRP-containing peptides were not cytotoxic to normal diploid human keratinocytes growing as primary cultures (Figure 2B) or to MRC5-hTERT immortalised fibroblasts (Figures 4A and 5A).

**Table 5:** Human in-vitro cancer cell lines showing a complete response to 200 μM THR53

Cell Line	Source	Histology
MGHU-1	ICR Sutton, Surrey	Carcinoma Bladder

H460	ATCC HTB-177NCI	Non-Small Cell Lung Carcinoma

SW620	ATCC CCL-227	Adenocarcinoma Colon

G361	ECACC 88030461	Melanoma

MCF7	ECACC 86012803	Adenocarcinoma Breast

H1299	ATCC CRL-5803	Non-Small Cell Lung Carcinoma

SK-BR-3	ATCC HTB-30	Adenocarcinoma Breast

A375	ATCC CRL-1619	Melanoma

U2OS	ATCC HTB-96	Osteosarcoma

SW480	ECACC 87092801	Adenocarcinoma Colon

RT112	ECACC 85061106	Carcinoma Bladder

### Time course and morphological changes of MRC5-hTERT and cancer cells exposed to 200 μM THR53

On time lapse photomicrography following exposure to THR53 (Figures 5B, C), dying RT112 and H460 cancer cells exhibited morphological features of increased vacuolation followed by swelling and disintegration, as opposed to the membrane blebbing and pyknosis which typify apoptosis. Progressive morphological changes in dying H460 cells exposed to 200 μM THR53 [additional file 1], contrasted with progressive cell division in untreated H460 cells [additional file 2] are shown as videos. Immortalised MRC5-hTERT fibroblasts, were unaffected by exposure to 200 μM THR53 except for some generalised slowing of growth (Figure 5A), [additional file 3] as compared to control, untreated, MRC5-hTERT cells [additional file 4]. RT112 cells were more sensitive to THR53 than H460 cells and died within 30 - 40 hours as compared to 96 hours for H460 and the majority of other human cancer cell lines investigated. For example H1299 human non-small cell lung cancer and MCF-7 breast cancer cells exhibited the same time course to death and morphological changes as H460 (Figures 4A and 4B).

### THR53 anticancer activity is dependent on cells being in cycle

**Figure 6:**
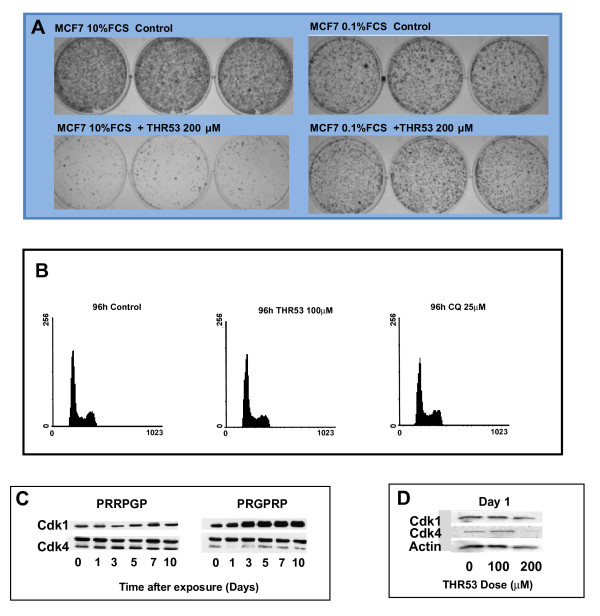
**THR53 cancer cell killing requires cells to have entered the cell division cycle, does not cause perturbation of cycling cells but does alter relative Cdk1/Cdk4 co-expression**. **A**. Macroscopic appearances of cycling THR53-treated MCF-7 human breast cancer cells in medium + 10% FCS (left hand panels) compared to the same cells held outside the cell cycle in medium + 0.1%FCS (right hand panel) **B**. DNA histograms of H460 human non-small cell lung cancer cells exposed to THR53 or an autophagy-inducing dose of 50 μM chloroquine. **C**. Sequential Western blots of Cdk1 and Cdk4 expression in RT112 human bladder cancer cells at 0 - 10 days after exposure to 5 mM linear, end-capped, PRGPRP. **D**. Western blot of Cdk1 and Cdk4 expression in H460 human non-small cell lung cancer cells 24 hours after exposure to 100 μM and 200 μM THR53.

THR53 had no effect on serum-deprived, MCF7 breast cancer cells (in medium + 0.1%FCS) whereas cycling MCF-7 cells in medium +10% FCS were killed (Figure 6A), indicating that MCF-7 cells needed to be in cycle to be killed. Cell death, however, was not dependent on cell division in MCF-7, RT112 or H460. THR53 had no effect on cell cycle phase distribution in H460 cells (Figure 6B) and PRGPRP hexapeptide cancer cell killing was unrelated to endogenous levels of Cdk4 or Cyclin D1. For example MGHU1 human bladder cancer and G361 human melanoma cells both exhibited total cell death with the same dose of 200 μM THR53 but respectively had markedly different endogenous Cdk4 values of 3.605 ± 0.735 and 6.035 ± 1.765 and Cyclin D1 values of 3.465 ± 0.832 and 1.055 ± 0.292 [24].

### The Cdk1/Cdk4 ratio changes following exposure to PRGPRP and THR53

Cdk1 and Cdk4 have been shown to have closely correlated expression across a wide range of human cancer cell lines [20]. This co-expression is unaffected by cytocidal doses of cisdiamminedichloroplatinum [24] but can accompany spontaneous cancer cell death [25]. Whether or not Cdk1/Cdk4 co-expression remained constant in cancer cells dying as a result of exposure to PRGPRP was thus examined by Western blotting. After exposure to PRGPRP, but not PRRPGP, RT112 cells showed a progressive rise over 10 days in Cdk1 expression compared to Cdk4 (Figure 6C). Similar results were obtained after exposure to 200 μM THR53 (Figure 6D), with complete disappearance of Cdk4 at 24 hours. These results suggest a potential role for PRGPRP in modulating Cdk4/Cdk1 co-expression in parallel with cancer cell killing and point to the possibility of the Cdk1/Cdk4 ratio as being a potential companion theranostic biomarker for PRGPRP anticancer activity.

### 100 μM THR53 causes depletion of ATP in dying H460 cells but does not induce autophagy

**Figure 7:**
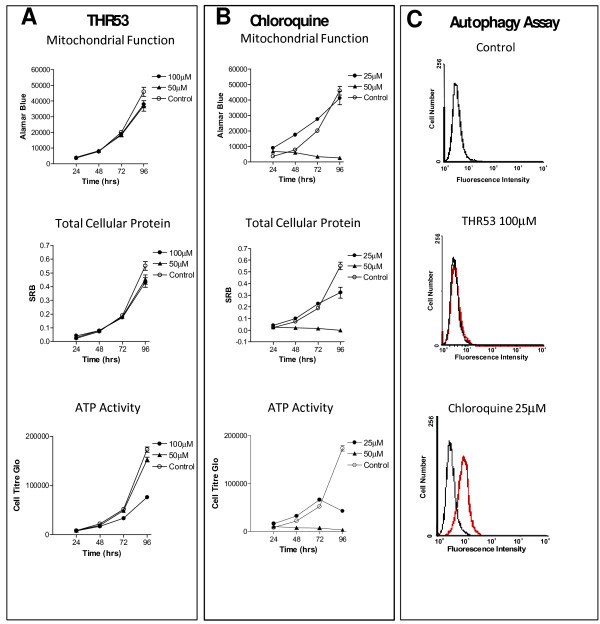
**Induction of changes in mitochondrial function, total cellular protein, ATP activity and autophagy in H460 human non-small cell lung cancer cells following exposure to THR53 or chloroquine**. **A**. Treatment with THR53 showing selective inhibition of ATP at 96 hours after initial exposure. **B**. Treatment of H460 with Chloroquine results in non-specific changes in all cell death parameters at 96 hours after initial exposure. **C**. THR53 treatment of H460 non-small cell lung cancer cells does not induce autophagy.

In order to probe the mechanism of PRGPRP analogue induced cell death, three assays were conducted on H460 human non-small cell lung cancer cells over a period of 96 hours following exposure to THR53 (Figure 7A). Total cellular protein was measured by sulphorhodamine B [31], mitochondrial oxidation/reduction was measured by Alamar Blue assay [32] and cellular ATP level was measured by luciferase activity [33]. A dose of 100 μM THR53 at which 50% of H460 cells were killed by 96 hours was chosen for assay comparison. Figure 7A shows progressive diminution of ATP levels between 48 and 96 hours after 100 μM THR53 without change in total cellular protein or altered mitochondrial oxidation/reduction. This selective ATP inhibition contrasts with the ubiquitous effect of chloroquine on all three parameters (figure 7B). Following exposure to THR53, morphological characteristics of apoptosis were not seen in the time lapse experiments, (Figures 5B and 5C) or in sub-G1 DNA content on cell cycle profile analysis; rather the cellular morphology of THR53 treated cells suggested death was either by necrosis or autophagy. An assay for autophagy was thus carried out which utilises a proprietary reagent (Cyto-ID Green) that specifically labels autophagolysosomes, co-localising with LC3, a specific autophagosome marker [34]. 25 μM Chloroquine was used as a positive control agent known to induce autophagy. In the cells stained with Cyto-ID Green, there was a clear rightward shift in the FL-1 peak at both 72 h and 96 h following chloroquine treatment, indicating an increase in autophagy in these cells. However, no difference in the mean fluorescent intensity was seen following treatment with 100 μM THR53 at either 72 h or 96 h, when compared with control.

### Trypan blue assay for necrosis in H460 and H1299 human non-small cell lung cancer cells and MCF-7 human breast cancer cells

The absence of autophagy in H460 cells indicates that this mode of cell death is unlikely to provide a general explanation for the selective lethality of PRGPRP compounds against human cancer cells. Necrosis is characterised by loss of cell membrane integrity. Viable cells do not incorporate trypan blue. Failure to exclude this dye reflects a loss of plasma membrane integrity associated with necrosis [35].

Figure 4A shows THR53 induces necrosis, as detected by trypan blue uptake, in all 3 human cancer cell lines but not in MRC5-h-TERT immortalised fibroblasts. The time courses of appearance of necrotic cells are closely similar for all three human cancer cell lines, being initially detectable at day 3 and progressively increasing over the following 4 days at comparable rates. In each case there is a parallel diminution in the percentage of viable cells. The ability to induce necrosis as identified by failure of trypan blue exclusion is dependent on the hexapeptide amino-acid sequence; being present with the amphiphilic cyclised polymer THR53 which carries PRGPRP but not with THR53C which carries PRRPGP (Figure 4C).

## Discussion

### Small peptides containing the PRGPRP amino-acid sequence are selectively toxic to a wide range of cancer cells whilst sparing normal cells

The amino-acid sequence Pro-Arg-Gly-Pro-Arg-Pro (PRGPRP) within small peptides is selectively cancerocidal towards a wide range of human in-vitro cancer cell lines but not normal diploid human keratinocytes, fibroblasts or immortalised MRC5-hTERT cells. The ubiquitous, selective anticancer activity is highly dependent on the arginines within the hexapeptide sequence, because alteration of the amino acid sequence to Pro-Arg-Arg-Pro-Gly-Pro removes the cancerocidal capacity as does substitution of the arginines for glutamic acid or L-NG-monomethyl-arginine. The lack of toxicity of PRGPRP compounds for non-cancerous cells and the removal of anticancer activity by modulation of the arginines in PRGPRP indicate that, although used here in high concentration, these compounds do have a truly selective cancerocidal activity. The relatively low specific activity of these peptides, at present, precludes their immediate progress to in-vivo studies as lead agents. Preliminary pharmacokinetic experiments with a maximum tolerated dose of THR53 (Institute of Cancer Therapeutics, University of Bradford, UK) have shown low plasma levels which were not persistent, indicating that there is a need for new PRGPRP congeners of higher specific activity. Having identified a novel therapeutic strategy and demonstrated proof of principle with the first and second generation compounds, medicinal chemistry studies to produce a further generation of compounds with improved PK are being undertaken.

The findings presented here of broad anticancer activity without toxicity to normal cells accompanied by a putative companion theranostic of altered Cdk1/Cdk4 ratios, however, merit continued investigation with the potential for development of a new range of anticancer therapeutics.

### PRGPRP-containing peptides could be causing cancer cell death by necrosis

Therapeutic stresses are generally understood to result in three possible types of programmed cell death (PCD); namely apoptosis (PCD I), autophagy (PCD II) or necrosis (PCD III) [36]. Following exposure to 200 μM THR53, the morphological appearances of two histologically distinct cancer cell lines, H460 and RT112 (Figures 5B, C) are not dissimilar to phase contrast images of Caco-2, human colon adenocarcinoma cells undergoing autophagic cell death 3 days after exposure to imiquimod [37]. There was, however, no evidence of autophagy in the Cyto-ID Green assay of H460 human lung cancer cells treated with a 100 μM dose of THR53 which causes 50% death in this cell line (Figure 7C). Moreover, morphological or DNA histogram manifestations of apoptosis were not seen after exposure to THR53. Apoptosis is an energy-requiring, cellular, autodestructive process dependent on ATP; in contrast to necrosis which is not ATP-dependent [36,38]. Depletion of ATP as seen in H460 cells treated with 100 μM THR53 would be expected to direct cell death towards necrosis rather than apoptosis. Moreover, H460 and two other cancer cell lines (H1299 and MCF-7) exhibited similar time courses for necrosis as assayed by trypan blue exclusion.

### Disruption of Cdk1/Cdk4 co-expression following exposure to 200 μM THR53

An extensive literature search has failed to provide an explanation for the relative elevation of Cdk1 in dying cancer cells following drug exposure and whether or not this might be related to necrotic cell death. Cdk1 elevation does, however, indicate that PRGPRP and its analogues are not apparently causing any defect in the classical Cdk4 downstream pathway. If proved consistent, however, such a change in Cdk1/Cdk4 co-expression could potentially provide a theranostic biomarker for PRGPRP analogue therapeutic efficacy.

### Potential protein-protein interactions involving PRGPRP

In keeping with its congruence to the PxxP motif described in binding to SH3 domains [39], PRGPRP is likely involved in protein-protein interactions. Singularly, however, the charged arginines of PRGPRP lie within, rather than flanking, the proline-rich core.

Despite the discovery route described here, however, and the intriguing location of PRGPRP within an externalised loop of Cdk4 not present in the homologous regions of Cdk2, Cdk6 or Cdk1, there is no direct evidence that this amino-acid sequence is functional within the whole normal Cdk4 protein. Resolving this will be the subject of future investigations. In addition, although it appears that the distance of the external loop containing the PRGPRP amino-acid sequence from the site of Cyclin D1 binding in the Cdk4 protein (Figure 1C) and the failure of conditional expression of the whole Cdk4 molecule to induce pRb phosphorylation (Figure 1B) or alter cell cycle phase distribution (Figure 6B), make it unlikely that PRGPRP itself and its congeners affect cyclin D1 binding and subsequent pRb phosphorylation, we have not directly confirmed this.

### Putative mechanism of PRGPRP cytotoxicity

The lack of efficacy of THR53 killing in quiescent MCF-7 breast cancer cells maintained in complete medium plus 0.1% FCS (Figure 6A), suggests that human cancer cells may need to have entered the cell cycle to become vulnerable to THR53. Nonetheless no perturbation of cell cycle phase distribution was seen. Based on all our currently available data, therefore, we hypothesise that, to supplement its classical cyclin-dependent kinase activity when bound to cyclin D, Cdk4 might also embody a kinase-independent signalling mechanism to release sufficient energy to enable cell division. Thus a putative function of the FPPRGPRPVQSV domain may be to promote the increase in ATP that is required to carry out cell division. A requirement for the FPPRGPRPVQSV region of Cdk4 to provide energy for cancer cell division could also provide an explanation for the fact that Cdk4 rather than Cdk2 or Cdk6 appears to be the mandatory cyclin-dependent kinase for carcinogenic malignant transformation.

Cancer cells in a metabolically active state of relentless cell division are highly energy dependent, deriving this energy from anaerobic metabolism as opposed to the aerobic metabolism of normal cells [40,41]. PRGPRP at high concentration may cause selective cancer cell necrosis by inhibition of anaerobic ATP provision, either as an isolated activity of presently unknown mechanism, or by competitive inhibition of the FPPRGPRPVQSV region of Cdk4. Disruption of energy provision, in this manner, might be expected to show the uniform selective cancer cell killing effect reported here. The detailed molecular interactions involved in these results require considerable further investigation. Nonetheless, ubiquitous, cancer-selective, killing by PRGPRP compounds may potentially herald new forms of anticancer therapy at the proteomic level.

## Materials and methods

Cell culture, clonogenic assays and Western blotting have previously been described [20,20]. All cell lines were from certified sources [24,30]. In addition to experiments in The Cancer Sciences Division of The University of Southampton some cell biology assays were carried out by Horizon Discovery Services (260 Cambridge Science Park, Milton Road, Cambridge, CB4 0WE). THR53, THR54 and THR79 (Synthesised by Polypeptide Laboratories Inc. 365 Maple Avenue Torrance CA 90503 USA) were prepared by dissolving in 10 μL of DMSO followed by introduction into 10 ml of tissue culture medium with rapid mixing, diluted to the required dose in tissue culture medium, introduced to the appropriate cells and left in situ for the duration of the experiment. Primary antibodies for Western blotting were: Cdk1 - mouse monoclonal sc-54 at 1/250, Cdk2 sc-748 at 1/250, Cdk4 - rabbit polyclonal sc-260 at 1/250, Cdk6 - rabbit polyclonal sc-260 at 1/250, pRb 105 rabbit polyclonal sc-50 at 1/1000, pRb 110 (hyperphosphorylated) rabbit polyclonal sc-32824 at 1/500 (All from Santa Cruz Biotechnology Inc, Bergheime Str 89-2, 69115 Heidelberg, Germany). The ecdysone-inducible *CDK4 *expression system in 2780 human ovarian carcinoma cells was constructed by cloning CDK4 cDNA from a previously described pcDNA3 vector [20] into a pIND vector (Invitrogen). Linearised constructs of this vector were transfected into 2780 cells with FuGENE™ reagent (Roche). Resistant colonies were selected by 2 weeks exposure to G418, expanded, and tested for increased Cdk4 expression following exposure to 1 μM muristerone.

**Mitochondrial function **(alamar blue) NCI-H460 cells were grown in Ham's F12 media supplemented with 10% FCS. Cells were harvested and added to 96-well plates at densities of 250-1000. Compounds were made up from stock solutions and added directly to cells at the concentrations indicated. Cells were grown with compound for the indicated time at 37°C, 5% CO2, in a humidified atmosphere. Alamar Blue 10% (v/v) was then added and incubated for a further 4 h, and fluorescent product detected using the BMG FLUOstar plate reader. The media only background was subtracted and the data analysed as appropriate.

**Total cellular protein **(sulphorhodamine B assay) was carried out on alamar Blue treated cells once this assay had been completed. Control medium/alamar blue were removed and 200 μl PBS was added. Cells were fixed by layering 25% (v/v) of a 50% TCA solution on top and incubating for 1 h at 4°C. Wells were rinsed with water and allowed to air dry. Cells were then stained in 0.4% SRB solution, before rinsing with 1% acetic acid and air drying. Incorporated dye was solubilised in 10 mM Tris and absorbance measured at 565 nm and 690 nm. Absorbance measurements at 690 nm were subtracted from the 565 nm readings to correct for multi-well plate absorbance.

**Cellular ATP levels **(CellTiter-Glo assay) Cells were grown, seeded and compound-treated as for the alamar Blue assay. A volume of the CellTiter-Glo reagent equal to the volume of cell culture media was added to the cells. Plates were mixed for 2 min on an orbital shaker to induce cell lysis before the plate was incubated at room temperature for 10 min to stabilize the signal. The luminescent signal was measured and the media only background was subtracted.

**Autophagy Assay **was performed as recommended by the manufacturers' instructions (Enzo ENZ-51031-K200). Briefly, H460 cells were seeded into 6 well plates at 2.5 × 10^5 ^and 1 × 10^5 ^cells/well. Immediately after cell plating, chloroquine or THR53 were added at the concentrations indicated. Following compound treatment, cells were washed once in PBS and resuspended in x1 Cyto-ID Green autophagy detection reagent and incubated at room temperature for 30 min. Immediately prior to analysis, propidium iodide (PI) solution was added to the cell suspension at a final concentration of 2.5 μg/ml, to assess cell death. Analysis was carried out using FACSCalibur (BD Biosciences). Cyto-ID Green autophagy reagent was measured in the FL-1 channel (530/30 nm bandpass filters with excitation at 488 nm) and PI measured in the FL-3 channel (670 nm longpass filters with excitation at 488 nm).

**Necrosis Assay **Cells were plated in triplicate in 96 well plates at concentrations of 500 cells per well (HT1299, MCF-7) or 1000 cells per well (H460, MRC5-hTERT) in the presence or absence of the relevant compound and assessed by Trypan Blue exclusion At 2,3,4 and 7 days after plating. To assay, cells were trypsinised off, stained with trypan blue in isotonic medium and live and dead cells separately counted visually by haemocytomter. Each data point is the mean of results from triplicate wells (Solid lines - Live cells, Broken lines - Dead Cells; Blue - Control, Red - THR53 200 μM).

### Structural Studies

The amino acid sequences used in this work were obtained from the Swiss-Prot and TrEMBL databases, maintained at the Expasy molecular biology server (http://ca.expasy.org/). The sequence similarity searches were performed using BLAST [42] maintained by the Swiss Institute for Biology (SIB), using default parameters unless otherwise stated. The sequences for potential templates were obtained from the PDB via a Blast search. Global multiple sequence alignments were performed using the program ClustalX [43]. Secondary structure alignments were performed using Swiss PDB-viewer v3.7 [44].

### Modelling Work

The x-ray crystal structures of Cdk6 and Cdk2 were obtained from the PDB. The suitability of the structures as templates was assessed by the program WHAT-CHECK [45] The program JACKAL [46] was used to build the homology model. The program Profix, a utility program distributed with JACKAL, was used to replace those residues and atoms missing from the structures. The models were constructed as follows:

1. Using the global multiple alignment, corrected for secondary structure, the program mutates non-conserved residues while retaining the original backbone conformation. The mutated residues were subjected to energy minimisation to remove atom clashes. The minimisation was performed in torsion angle space, using the fast torsion angle minimiser implemented in JACKAL. The energy function uses the CHARMM22 all atom force field [47]. Insertions and deletions are then performed, with the bonds closed using a random tweak method. The results are again minimised.

2. The secondary structure was assigned using a DSSP-like routine [48].

3. Prediction of the identified loop regions was performed as follows: a) First, the original backbone segment was deleted and replaced by a new segment that was made by generating a large number of random backbone conformations, which were then closed using a random tweak method, b) the closed conformers were subjected to energy minimisation using the fast torsion angle minimiser, c) the side chains were then modelled using a large rotamer library of 3222 rotamers in 10° bins and subjected to further minimisation, d) the best candidate, the conformer with the lowest energy, was retained, and a further round of conformation sampling was performed about the new conformation, e) the final structure was subjected once again to energy minimisation.

4. The secondary structure elements were then refined by again sampling through a backbone rotamer library, but with the original rotamer retained in the sampling. To retain the hydrogen-bonding network of the existing secondary structure, a large energy penalty was incurred by any conformation that broke an existing hydrogen bond (hydrogen bonds are defined as in DSSP). The lowest energy conformation was retained. The side chains were then built in a similar way.

5. The final model was minimised using the torsion angle minimiser.

6. After the construction of the model, it was subjected to 500 steps of steepest descent full energy minimisation using AMBER, with the parm96 force field [49]. The polar hydrogen atoms were added by WHATIF [50] after optimising the hydrogen-bond network.

7. Steps 1-6 were repeated until no further improvement in the model was obtained.

At points it was also necessary to manually tweak the structures. This was performed through the Swiss PDB-viewer. The final models were assessed for accuracy and quality by the programs WHAT-CHECK and Swiss PDB-viewer. The threading energy given by Swiss PDB-viewer is based on the potential of mean force developed by Sippl et al. [51]. The molecular mechanics energy was calculated using the GROMACS96 force field [52] and was also implemented in Swiss PDB-viewer.

## Declaration of Competing interests

HMW is currently CEO of HilRos Ltd and Founder of Theryte Ltd in which he has a shareholding. Until March 2009 he was CMO and Director of R&D for Theryte Ltd. HMW JDK and JWE are the authors of patents pertinent to the work described in this paper. The patents are held by Theryte Ltd. but no authors have received personal remuneration from Theryte Ltd within the past 5 years. Theryte has, in the past, funded chemical and biological studies in the University of Southampton including the salary of UA. Some experiments investigating potential mechanisms of cell death were outsourced to Horizon Discoveries and financed personally by HMW via HilRos. The article processing charge is distributed amongst the authors. The remaining authors declare that they have no competing interests.

## Authors' contributions

HMW was responsible for writing the manuscript, the original concept of a potential non-kinase functional site unique to Cdk4 which prompted structural comparison of the C'-terminal regions of Cdks 2, 4 and 6, identifying the biological effects of PRGPRP on cancer and normal cells accompanied by changes in Cdk1/Cdk4 co-expression and the design, initial testing and clonogenic assays of the cyclic PRGPRP analogues THR53 and THR54. JDK, JWE and RIM carried out the structural chemistry modelling and pinpointed the FPPRGPRPVQSV region unique to Cdk4. JPB and UA demonstrated morphological changes induced by THR53 by plain and time-lapse photomicrography, identified the early effects of this peptide on Cdk4 by Western blotting, carried out clonogenic assays on a range of human in-vitro cancer cell lines, showed that MCF7 cells which were not in cycle were unaffected by THR53 and carried out trypan blue exclusion assays for necrosis. LS showed co-expression of Cdk1 with Cdk4 but not with Cdk2 or Cdk6 in human cancer cell lines and co-elevation of endogenous Cdk1 with exogenous Cdk4 in the absence of pRb phosphorylation in muristerone-inducible 2780 pvgrPIND cells.

All authors have read and approved the final manuscript.

## Supplementary Material

Additional file 1 **Time Lapse Photomicrographic Video of H460 human non-small lung cancer cells exposed in-vitro to 200 μM THR53**. H460 cells were grown in the presence of 200 μM THR53 in Ham's F12 medium + 10% FCS. Micrographic video frames at ×40 magnification were taken at 20 minute intervals and are shown at 7 frames per second. In contrast to control H460 cells, grown in the same medium in the absence of THR53 (see below), although undergoing repeated cell divisions, treated H460 cells show progressive cell death throughout succesive divisions, typified by cellular swelling, vacuolation and disintegration. There is no morphological evidence of apoptosis. Click here for file

Additional file 2 **Time Lapse Photomicrographic Video of H460 human non-small lung cancer cells growing in normal medium in-vitro in the absence of THR53**. H460 cells were grown in Ham's F12 medium + 10% FCS without THR53. Micrographic video frames at ×40 magnification were taken at 20 minute intervals and are shown at 7 frames per second. Control H460 cells, undergo normal repeated cell divisions without evidence of cell death. Click for file

Additional file 3 **Time Lapse Photomicrographic Video of MRC5-hTERT immortalised normal human fibroblasts exposed in-vitro to 200 μM THR53**. MRC5-hTERT immortalised normal human fibroblasts were grown in the presence of 200 μM THR53 in Ham's F12 medium + 10% FCS. Photomicrographic video frames at ×40 magnification were taken at 20 minute intervals and are shown at 7 frames per second. In contrast to H460 cells exposed to THR53, MRC5-hTERT cells although showing slight slowing in their division rate compared to untreated MRC5-hTERT cells, progressed through a series of cell divisions without any evidence of cell death. Click here for file

Additional file 4 **Time Lapse Photomicrographic Video of Control MRC5-hTERT immortalised normal human fibroblasts growing in the absence of THR53.THR53**. Control MRC5-hTERT immortalised normal human fibroblasts were grown in the absence of THR53. Photomicrographic video frames at ×40 magnification were taken at 20 minute intervals and are shown at 7 frames per second. MRC5-hTERT immortalised normal human fibroblasts went through a series of repeated cell divisions without evidence of cell death. Click here for file
